# OCT4B1 Regulates the Cellular Stress Response of Human Dental Pulp Cells with Inflammation

**DOI:** 10.1155/2017/2756891

**Published:** 2017-04-04

**Authors:** Lu Liu, Rong Huang, Ruiqi Yang, Xi Wei

**Affiliations:** ^1^Operative Dentistry and Endodontics, Guanghua School of Stomatology, Affiliated Stomatological Hospital, Guangdong Province Key Laboratory of Stomatology, Sun Yat-Sen University, Guangzhou, Guangdong, China; ^2^Institute of Health and Biomedical Innovation and Science and Engineering Faculty, Queensland University of Technology, Brisbane, QLD, Australia

## Abstract

*Introduction.* Infection and apoptosis are combined triggers for inflammation in dental tissues. Octamer-binding transcription factor 4-B1 (OCT4B1), a novel spliced variant of OCT4 family, could respond to the cellular stress and possess antiapoptotic property. However, its specific role in dental pulpitis remains unknown.* Methods.* To investigate the effect of OCT4B1 on inflammation of dental pulp cells (DPCs), its expression in inflamed dental pulp tissues and DPCs was examined by in situ hybridization, real-time PCR, and FISH assay. OCT4B1 overexpressed DPCs model was established, confirmed by western blot and immunofluorescence staining, and then stimulated with Lipopolysaccharide (LPS). Apoptotic rate was determined by Hoechst/PI staining and FACS. Cell survival rate was calculated by CCK8 assay.* Results.* In situ hybridization, real-time PCR, and FISH assay revealed that* OCT4B1* was extensively expressed in inflamed dental pulp tissues and DPCs with LPS stimulation. Western blot and immunofluorescence staining showed the expression of OCT4B1 and OCT4B increased after OCT4B1 transfection. Hoechst/PI staining and FACS demonstrated that less red/blue fluorescence was detected and apoptotic percentage decreased (3.45%) after transfection. CCK8 demonstrated that the survival rate of pCDH-OCT4B1-flag cells increased.* Conclusions.* OCT4B1 plays an essential role in inflammation and apoptosis of DPCs. OCT4B might operate synergistically with OCT4B1 to reduce apoptosis.

## 1. Introduction

The dental pulp tissue is surrounded by inelastic pulp chamber resulting in rapid tissue edema and necrosis in response to inflammation. Due to lacking of effective collateral circulation, bacteria induced dental pulpitis is difficult to reverse. Dental pulp cells (DPCs), a heterogeneous cell population, contain progenitor/stem cells with self-renewal, colony-forming efficiency, and multilineage differentiation capability. DPCs are able to differentiate into odontoblasts, chondrocytes, adipocytes, neurons, and so forth. DPCs are hopeful cell source for dental tissue regeneration [1, 2].

Considerable evidence shows that the suboptimal regenerative outcomes are associated with infections and necrosis mediated by inflammatory responses [3]. For bone regeneration, a mild immune response is necessary in the early stage of regeneration to initiate the healing cascade. If the immune response stays mild and is resolved promptly, tissue regeneration occurs. Nevertheless, once healing tissues become infected and the inflammatory response persists, an adverse effect on bone regeneration occurs [4–7]. Correspondingly, dental pulp exposed to bacteria may become inflamed and irritated by Lipopolysaccharide (LPS). Exposure to LPS activates the cellular stress response, which results in apoptosis and cell death in dental pulp. Pathological alternations of DPCs caused by LPS could give rise to acute dental pain and adversely affect the repair process [8–10]. Therefore, it is of great importance to develop the anti-inflammatory strategy during dental regeneration.

Octamer-binding transcription factor 4 (OCT4, OTF3, and OTF4), belonging to the POU (Pit-Oct-Unc) family, consists of numerous transcription factors owning the same POU domain. Most of the transcription factors take part in early embryogenesis and exert the regulation property in lineage specific differentiation and neural development [11]. Like other members, OCT4 has different connecting regions and two conservative subdomains, which can interact with DNA to activate or inhibit the expression of target genes [12]. Human* OCT4* gene locates on chromosome 6 and contains 5 exons [13]. It consists of three splicing variants, termed OCT4A (variant 1, NM_002701), OCT4B (variant 2, NM_203289), and OCT4B1 (variant 3, EU518650). OCT4B1 is mainly expressed in pluripotent cells, albeit being barely expressed in differentiated cells, indicating that it might be an essential marker for stem cells [14, 15]. It also contributes to tumorigenesis of gastric cancer and colorectal cancer [16, 17]. More importantly, OCT4B1 was able to encode the same protein isoforms of OCT4B since OCT4B1 mRNA could be spliced into OCT4B mRNA during transcription. The splicing can be inhibited by the mutation of the classical splicing site from OCT4B1, suggesting that OCT4B may originate from OCT4B1 during mRNA alternative splicing [18]. So far, the relationship between OCT4B1 and OCT4B, as well as their roles in inflammation, remains unknown. Therefore, the present study is to identify the effect of Oct4B1 on the antiapoptotic performance of DPCs in response to inflammation, in order to elucidate the relationship between dental inflammation and regeneration.

It is reported that OCT4B1 plays an antiapoptotic role in cancer cells, which might be associated with cellular stress [16, 19, 20]. Our previous studies revealed that OCT4 was expressed in the early passages of DPCs and its expression decreased during cell differentiation [21]. We also demonstrated that OCT4 expression was upregulated three times under the ischemia condition in vitro [22]. Here we extended our investigation to the effect of the splicing variant OCT4B1 on the antiapoptotic property of DPCs in response to inflammation, in order to elucidate the relationship between inflammation and regeneration.

## 2. Materials and Methods

### 2.1. Tissue Preparation and Cell Culture of DPCs

Extracted wisdom teeth including those diagnosed as irreversible pulpitis were collected after obtaining informed consent from every patient (18–25 years old). Ethics approval for this study was granted by the Ethics Committee of Sun Yat-Sen University. The teeth were split along the developmental groove and fixed with 4% paraformaldehyde for 24 h. They were then decalcified, embedded in paraffin, and prepared into 4 mm thick serial sections. All the surgical instruments and glass slides used for RNA detection were treated with diethylpyrocarbonate (DEPC, Beyotime, Guangzhou, China) to inactivate RNase enzymes. DPCs were cultured as previously described [23]. Briefly, DPCs were cultured in Dulbecco's modified Eagle medium with low glucose (DMEM-LG, Invitrogen, CA, USA) supplemented with 10% fetal bovine serum (FBS, HyClone, UT, US), 10 U/mL penicillin, and 10 *μ*g/mL streptomycin (Invitrogen, CA, USA), and incubated at 37°C in 5% CO_2_. The medium was changed every 3 days. The cells were passaged using 0.25% trypsin/EDTA (Life Technologies, Carlsbad, California, USA).

### 2.2. In Situ Hybridization

The digoxin and fluorescent probes (Biosense, Guangzhou, China) were synthesized with the following sequence: 5′-CCATTCGGGATTCAAGAACCTAC-3′. 6-Carboxyfluorescein (6-FAM) was labeled at the 3′ terminal for the latter. Paraffin sections were first stained with hematoxylin-eosin (HE) and divided into two groups according to the pathological changes. All the sections were dewaxed, dehydrated, and digested by pepsin at 37°C for 30 min. After 2 h before hybridization, dental pulp tissues were hybridized with the digoxin probe at 37°C overnight. Biotin-labeled digoxin antibody, streptavidin–biotin–peroxidase complex (SABC), and biotinylated peroxidase were added dropwise. Sections were developed with DAB and hematoxylin and then mounted with neutral gum.

### 2.3. Expression of OCT4B1 in DPCs with LPS Treatment

For OCT4B1 detection, DPCs were seeded in a 6-well plate with 5 × 10^5^ cells/well. After 24 h incubation, the culture medium was removed and cells were stimulated with 100 ng/mL LPS derived from* Escherichia coli* 0111:B4 (Sigma, Missouri, USA) as previously described [7]. DPCs were then fixed for 20 min and permeabilized with 0.5% Triton X-100 for another 15 min. After ethanol dehydration and probe degeneration, hybridization buffer mixed with the probe was added and hybridized overnight at 37°C. After being washed with 50% formamide and 2x Standard Saline Citrate (SSC), the cells were counterstained with DAPI (1 : 1400) and observed under confocal laser scanning microscopy (Carl Zeiss, Germany).

DPCs were stimulated with 100 ng/m LPS for 24, 48, and 72 h, respectively. Total RNA was extracted by TRIzol reagent (Life Technologies, CA, USA) for mRNA detection. One microgram of total RNA was used for reverse transcription into cDNA (Transcription First Strand cDNA Synthesis Kit, Roche, Rotkreuz, Switzerland) and twenty microliter was used for PCR amplification (SYBR Green I Master, Roche, Switzerland). The primers in this experiment were listed as follows: OCT4B1 (forward: 5′-GGGTTCTATTTGGTGGGTTCC-3′, reverse: 5′-TCCCTCTCCCTACTCCTCTTCA-3′); *β*-actin (forward: 5′-AGTGGGTTGTTTGCCTTTGG-3′; reverse: 5′-TCGTCCCAGTTGGTGACGAT-3′). The whole process of amplification was 40 cycles and repeated denaturing at 95°C, annealing at 55°C, and extension at 72°C. The data were analyzed by 2^−ΔΔCt^ method.

### 2.4. Lentiviral Transfection of OCT4B1 in DPCs and Western Blot

Since there is no specific antibody for OCT4 isoform OCT4B1, we designed, produced, and characterized OCT4B1 with a FLAG tag embedded upstream of the RGD motif. Briefly,* OCT4B1* was amplified by PCR and cloned into pCDH-CMV-MCS-EF1-GFP and pCDH-CMV-MCS-EF1-puro lentiviral vector (Dakai Biotech. Co., Guangzhou, China). The flag octa peptide was bound to the plasmid for the sake of generating fusion protein of OCT4B1, psPAX, and pMD2.* Lentivirus* packaging system and the plasmid were used to transfect 293T cells. After 48 h incubation, the supernatant was harvested and lentivirus titer was obtained. The virus liquid was then used to infect DPCs. The optimal multiplicity of infection (MOI) was determined. The recombinants generated with OCT4B1, flag, or GFP genes were termed pCDH-OCT4B1-flag-GFP, pCDH-GFP, pCDH-OCT4B1-flag, and pCDH, respectively. The nontransfected DPCs served as control.

DPCs were harvested with sodium dodecyl sulfate (SDS) lysis buffer, sonicated, and centrifuged at 12,000*g* for 10 minutes. The total protein in cell extract was measured by Pierce BCA Protein Assay Kit (Thermo Scientific, USA). Twenty micrograms of protein was separated by sodium dodecyl sulfate-polyacrylamide gel electrophoresis (SDS-PAGE) and transferred to a nitrocellulose membrane using 200 mA for 1 h. The nitrocellulose membrane was blocked in 5% nonfat milk for 1 h at room temperature, rinsed, and incubated with anti-OCT4B antibody (1 : 1000, Bioss, MA, USA) and anti-tag antibodies (DDK antibodies) (1 : 4000, Origene, MD, USA) overnight at 4°C. After washing three times, the membrane was incubated with the donkey anti-goat IgG-HRP and goat anti-mouse IgG-HRP (1 : 4000, Santa Cruz, CA, USA) at room temperature for 1 hour. Novex ECL Chemiluminescent Substrate Reagent Kit (Life Technologies, CA, USA) was used to collect the luminescent signals.

### 2.5. Immunofluorescence Staining of FLAG Tag in DPCs

DPCs were seeded and fixed on cell slides. All the slides were blocked in 3% BSA for 30 minutes at room temperature. Mouse monoclonal anti-DDK antibody (1 : 4000, Origene, MD, USA) was used for detection of flag tag. Alexa Fluor® 647 conjugated anti-mouse IgG fragments (1 : 1000, Cell Signaling Technology, MA, USA) staining was performed for 1 h at room temperature. The stained cells were observed under the confocal laser scanning microscopy (Carl Zeiss, Germany).

### 2.6. Apoptosis and Necrosis Analysis

DPCs were cultured in 24-well plates and stimulated with 100 ng/mL LPS for 24 h. The cells were stained with Hoechst 33342/PI Double Staining Kit (Keygen Biotech Co., Nanjing, China) for 10 min at room temperature and observed under the inverted fluorescence microscope. For fluorescence activated cell sorting (FACS) analysis, cells were digested with EDTA-free trypsin and stained with Hoechst 33342/PI at room temperature for 10 minutes. Blue fluorescent of Hoechst 33342 and red fluorescence of PI were captured with Gallios Flow Cytometer (Beckman coulter, CA, USA).

### 2.7. Cell Viability Analysis

DPCs were seeded in 6-well plates and starved in serum-free medium overnight. The result of LPS cytotoxicity was examined by Cell Counting Kit-8 (CCK-8, Jingxin, Guangzhou, China). The optical density (OD) value was assayed to calculate the final cell number. Cell survival rate was calculated as follows: cell viability (%) = (cell number (LPS) − cell number (blank))/(cell number (without LPS) − cell number (blank)) × 100.

### 2.8. Statistical Analysis

All experiments were repeated at least three times. The SPSS19.0 software package (SPSS Inc., Chicago, IL) was used for the statistical tests. All the data were analyzed using one-way ANOVA analysis and Student's *t*-test. The difference was considered as being of statistical significance at *P* < 0.05.

## 3. Results

### 3.1. OCT4B1 Expression in Dental Pulp with Inflammation

**Figure 1:**
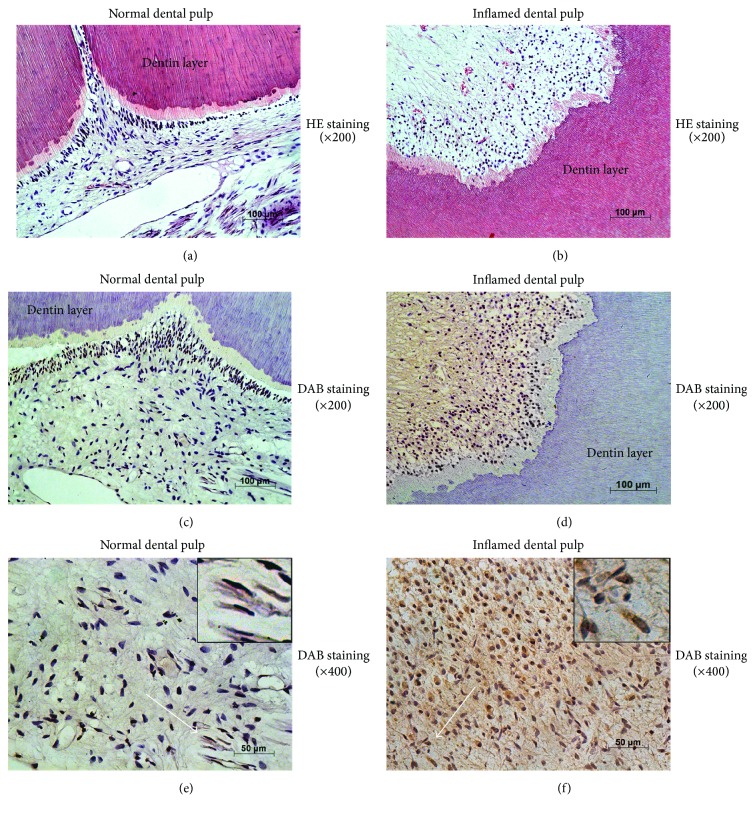
Expression of OCT4B1 in the dental pulp tissues with inflammation. (a) HE indicated that odontoblasts lined along the pulp-dentin border below which the tubular dentin was regularly presented in noninflamed pulp (×200). (b) Odontoblasts were disorderly arranged with inflammatory cell infiltration in the inflammatory pulp-dentin complex (×200). ((c) and (e)) In situ hybridization assay revealed that, in the noninflamed dental pulp tissues, OCT4B1 (white arrows) was weakly expressed in the cytoplasm of DPCs (×200, ×400), whereas OCT4B1 was expressed in both the cytoplasm and nucleus of the inflamed DPCs ((d) and (f), ×200 and  ×400).

The structure and pathological states of dental pulp tissues were detected by HE staining. Odontoblasts lined along the pulp-dentin border below which the tubular dentin was regularly presented in noninflamed dental pulp (Figure 1(a)). In inflamed dental pulp tissues, odontoblasts were surrounded by locally infiltrated immune cells and disorganized tubular dentin was observed beneath the odontoblasts layer (Figure 1(b)). In situ hybridization assay revealed that, in the noninflamed dental pulp tissues, OCT4B1 was weakly expressed in normal DPCs (Figures 1(c) and 1(e), ×200 and  ×400), whereas OCT4B1 was extensively expressed in both the cytoplasm and nucleus of the inflamed DPCs (Figures 1(d) and 1(f), ×200 and  ×400).

### 3.2. Expression of OCT4B1 in DPCs with LPS Treatment

**Figure 2:**
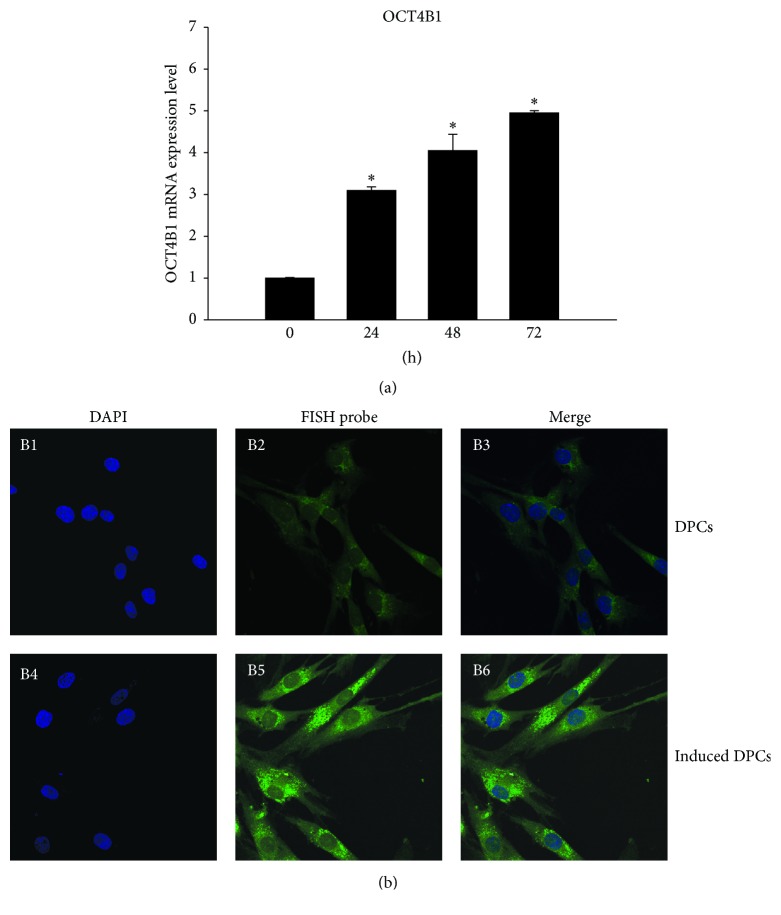
Expression of OCT4B1 in DPCs with LPS treatment. (a) Real-time PCR demonstrated that the expression level of* OCT4B1* mRNA was elevated significantly over time (^*∗*^*P* < 0.05). (b) (B1–B3) FISH assay showed that green fluorescence representing OCT4B1 was weakly observed in the cytoplasm of normal DPCs (×400). (B4–B6) Green fluorescence representing that OCT4B1 was strongly expressed in the cytoplasm around nucleus of DPCs after 72 h LPS stimulation (×400).

The result of real-time PCR revealed that* OCT4B1* mRNA expression in DPCs increased significantly over time (*P* < 0.05) (Figure 2(a)). FISH assay showed that punctiform green fluorescence was weakly expressed in the cytoplasm of normal DPCs (Figure 2(b), B1–B3), whereas the green fluorescence was enhanced and gathered in the cytoplasm around nucleus of DPCs after 72 h LPS stimulation (Figure 2(b), B4–B6). Both of the results indicated the correlation between OCT4B1 and LPS-induced inflammation.

### 3.3. Expression of OCT4B1 in DPCs after Lentiviral Transfection

**Figure 3:**
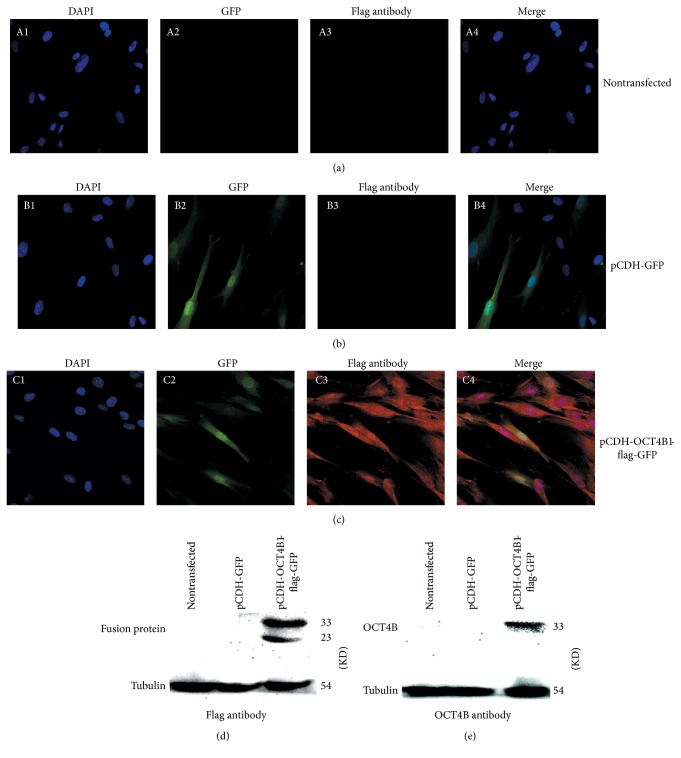
Expression of OCT4B1 in DPCs with lentiviral transfection. Green fluorescence was observed in the nucleus and cytoplasm of both pCDH-OCT4B1-flag and pCDH-flag cells. Red fluorescence representing flag octa peptide was expressed only in the nucleus and cytoplasm of pCDH-OCT4B1-flag-GFP cells. (a) (A1–A4) No green or red fluorescence was expressed in nontransfected cells (×400). (b) (B1–B4) Blue and green fluorescence were expressed in pCDH-GFP cells (×400). (c) (C1–C4) Blue, green, and red fluorescence could be observed in the OCT4B1 overexpression cells (×400). (d) Western blot showed that no protein band was detected in nontransfected pCDH-GFP cells. 23 KD (OCT4B1) and 33 KD (OCT4B) protein bands were detected with flag antibody. (e) There was a blurred band of 33 KD detected with OCT4B antibody, demonstrating that the expression of OCT4B was increased after OCT4B1 transfection.

Green fluorescence was observed in the nucleus and cytoplasm of both pCDH-OCT4B1-flag (Figure 3(c), C2) and pCDH-flag cells (Figure 3(b), B2). Red fluorescence representing flag octa peptide was expressed only in the nucleus and cytoplasm of pCDH-OCT4B1-flag-GFP cells (Figure 3(c), C3), indicating the successful induced expression of OCT4B1 in DPCs after transfection. Neither green nor red fluorescence was expressed in nontransfected cells (Figure 3(a), A2–A4). The results of western blot agreed with the above results; no protein band was detected in nontransfected pCDH-GFP cells. 23 KD (OCT4B1) and 33 KD (OCT4B) protein bands were detected with OCT4B1 flag antibody (Figure 3(d)). There was a blurred band of 33 KD detected with OCT4B antibody, demonstrating that the expression of OCT4B was increased after OCT4B1 transfection (Figure 3(e)).

### 3.4. Effect of OCT4B1 in DPCs with LPS Induction

**Figure 4:**
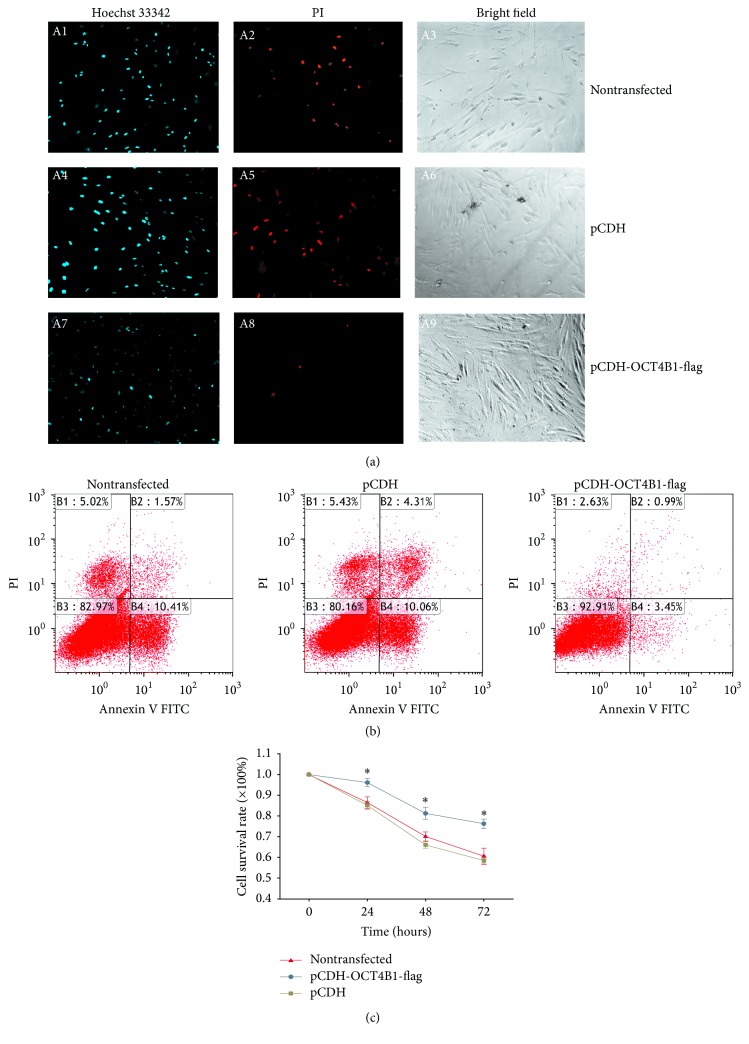
Effect of OCT4B1 in DPCs with LPS induction. (a) The nontransfected, pCDH, and pCDH-OCT4B1-flag DPCs were treated with 100 ng/L LPS for 24 h and then stained with Hoechst 33342 and PI. Dark blue represented apoptotic cells and red fluorescence represented dead cells. The total cell number was observed under the bright field (×100). Cell number of apoptosis and death of pCDH-OCT4B1-flag cells (A7–A9) was remarkably lower than that of the nontransfected (A1–A3) and pCDH groups (A4–A6). (b) Cell apoptotic rate was examined by FACS. The percentage of apoptotic cells was decreased (3.45%) in DPCs with OCT4B1 overexpression compared with the nontransfected and pCDH DPCs (10.41% and 10.06%) (*P* < 0.05). (c) The line graph showed that, after being induced with LPS for 72 h, the survival rate of pCDH-OCT4B1-flag cells was significantly higher than nontransfected and pCDH DPCs (^*∗*^*P* < 0.05).

Plasmids without GFP were used to avoid spectral cross of green fluorescence in this experiment. Cells with or without lentiviral transfection were stimulated with 100 ng/mL LPS for 24 h. Hoechst/PI staining showed less red fluorescence and blue fluorescence was detected in DPCs with OCT4B1 overexpression (Figure 4(a), A8) compared with nontransfected (Figure 4(a), A2) and pCDH DPCs (Figure 4(a), A5). The results of FACS showed the percentage of apoptotic cells was decreased (3.45%) in DPCs with OCT4B1 overexpression compared with the nontransfected and pCDH DPCs (10.41% and 10.06%) (Figure 4(b)). The line graph showed the survival rate of pCDH-OCT4B1-flag cells was significantly higher than nontransfected and pCDH DPCs (*P* < 0.05) (Figure 4(c)).

## 4. Discussion

OCT4 is a crucial stemness marker expressed in embryonic stem cells (ESCs) and embryonic carcinoma cells (ECCs). In 2006, Takahashi and Yamanaka generated the induced pluripotent stem (iPS) cells from fibroblasts by introducing four factors, OCT4, Sox2, c-Myc, and Klf4 [24]. The follow-up study confirmed that a single factor OCT4 could induce the generation of iPS cells [25]. The inner cell mass was not able to maintain the pluripotency in* OCT4* gene knockout mice [26], which highlighted the important role of OCT4 in cell reprogramming. Study revealed that knocking out* OCT4* with Cre/Loxp system in primordial germ cells (PGCs) led to apoptosis of PGCs rather than differentiation into trophectodermal lineages [27], providing the evidence of the functional diversity of OCT4.

The discovery of OCT4B1 ignited the hot investigation into OCT4 family. The initial study of OCT4B1 demonstrated that it was extensively expressed in human ESCs and ECCs and downregulated during differentiation [14]. It was also expressed in nonpluripotent cells such as gastric adenocarcinoma, bladder tumor, and colorectal cancer cells, as well as normal adult stem cells [16, 17, 20]. OCT4B1 expression in bladder cancer cells increased when heated up at 45°C [18]. Interfering with OCT4B1 siRNA, the morphology and cell cycle of gastric adenocarcinoma cells (AGS) were altered remarkably, suggesting the potential functional change of AGS [19]. Suppression of OCT4B1 in several tumorous cell lines resulted in upregulation of proapoptotic genes and downregulation of antiapoptotic genes, which implies that OCT4B1 might affect apoptotic rate through several stress related pathways [28]. Therefore, OCT4B1 may play an important role in the regulation of cellular stress. Moreover, OCT4B1 expression levels were elevated in both tissue and blood samples from patients with inflammatory bowel disease as a result of tissue damage, indicating a possible role of OCT4B1 played in anti-inflammation and regeneration [29]. In the present study, the ISH and FISH assay indicated that* OCT4B1* expression was elevated in the inflamed dental pulp and in DPCs with LPS induction, suggesting that OCT4B1 might be involved in the cellular stress.

OCT4 is a transcriptional factor functioning in nucleus. However, the splicing variant OCT4B1 was reported to be expressed in the cytoplasm of cancer cells [19]. Our previous data showed that the activity of OCT4 in PDLCs was associated with pluripotency and multilineage differentiation potential, which might be controlled by nucleus-cytoplasmic shuttling [30]. In the present study, we found that OCT4B1 was mainly located in the cytoplasm around nucleus of DPCs after LPS treatment, implying the potential nucleus-cytoplasm shuttling of OCT4B1 in inflamed DPCs, whereas OCT4B1 fusion protein was expressed in both nucleus and cytoplasm of DPCs after transfection. These results may be contributed to the complicated roles of OCT4, since initiation of translation usually generates functional diversity through regulation of the localization of protein isoforms. When the cytoplasmic location is detected, the nuclear envelope is disassembled, allowing proteins swap between nucleus and cytoplasmic compartments [30]. It might be possible that OCT4B1 was firstly located in nucleus, then translocated into cytoplasm, and functioned as an inflammation regulator. Thus the translocation of OCT4B1 from the nucleus to the cytoplasm may reflect its potential alternation of cell characteristics in response to inflammation.

OCT4A is highly concentrated in the nucleus of cells during human preimplantation development. Compared with OCT4A, OCT4B encodes a diverse N-terminal domain (NTD) at the start of the protein, localized mainly in the cytoplasm of most nonpluripotent cells and somatic cells [31, 32]. The electromobility shift assay revealed that the N-terminal domain (NTD) of OCT4B inhibits the binding of OCT4 to the DNA and prevents activation of transcription related genes. Therefore, OCT4B cannot sustain the pluripotency and self-renewal of ES (embryonic stem) cells and overexpression of OCT4B cannot activate transcription from OCT4-dependent promoters [33]. Researchers used different primer sets for OCT4B isoform and found that ES and EC cells consistently expressed another variant of OCT4, named OCT4B1. OCT4B1 is highly expressed in ESCs and ECCs (embryonic carcinoma cells) and is rapidly downregulated upon induction of differentiation. Based on their study on stage specific embryonic antigen 3 positive (SSEA3+) stem cells, there is a potential relationship between the expression of OCT4B1 and the undifferentiated state of ES cells [30]. However, so far no evidence shows the localization alternation of OCT4B1 and its potential correlation with OCT4A and OCT4B. Thus in the present study, we examined the expression of OCT4B after OCT4B1 lentiviral transfection. Expression of OCT4B was elevated along with upregulation of OCT4B1. The results showed that OCT4B1 might encode OCT4B. This can be conceived as there was an overlap in the translation process of OCT4B1 and OCT4B. It is possible that a certain amount of* OCT4B1* mRNA was alternatively spliced into* OCT4B* mRNA after lentiviral transfection. This is in accord with the report of OCT4B1 in tumor cell lines [18]. Therefore, we provided the evidence that OCT4B1 might be the precursor of OCT4B.

It is reported that the apoptotic rate of Hela cells with OCT4B overexpression was downregulated after being induced at 45°C for 2 h, suggesting that OCT4B had an antiapoptotic property in response to cellular stress [34]. However, whether this function was associated with OCT4B1 remains unknown. It is also demonstrated that downregulation of OCT4B1 could enhance the activity of caspase-3 and caspase-7 in AGS and the number of apoptotic cells significantly increased compared with the nontreated cells [16]. In our study, the cell viability was remarkably upregulated in DPCs with OCT4B1 overexpression under inflammatory conditions. This is probably because OCT4B1 exert an antiapoptotic effect on DPCs and may be attributed to the maintenance of cell survival. Moreover, OCT4B1 may convert into OCT4B to perform the antiapoptotic function.

So far, there is no specific antibody for OCT4 isoform OCT4B1, which makes it difficult for researchers to study the distinct potential roles of OCT4 isoforms in regeneration and inflammation. In the present study, we designed, produced, and characterized OCT4B1 with a FLAG tag embedded upstream of the RGD motif. Moreover, the embedded FLAG tag was recognized by anti-FLAG (DDK) antibodies, which allowed OCT4B1-FLAG to be distinguished by western blot assay. Epitope tags encoded in DNA sequences provide a way to localize genes in various cell types and characterize newly identified, low abundance, or poorly immunogenic proteins without specific antibodies. The commercially available antibodies to epitope tags (DDK antibodies) are developed to optimize the detection and purification of the recombinant proteins. DDK antibodies with high sensitivity and specificity are commercially produced and widely used in various literatures. Thus epitope tags are an attractive approach to proteins without further requirement of generation and validation of protein-specific antibodies [35, 36]. Therefore, the knowledge generated from the present study might shed light on the methodology to explore the potential roles of OCT4 isoforms in the future study.

Taken together, the present study revealed that OCT4B1 is closely related to the inflamed dental pulp and possesses an antiapoptotic property through regulation of OCT4B. OCT4B1 might be one of the antiapoptotic candidates for pulpitis. However, further studies are needed to investigate the underlying mechanism of OCT4B1 in apoptosis and its potential role in dental regeneration.
